# Prospective and Retrospective Study of PPR in Goats: Epidemiology, Clinical Signs, Risk Factors, and Hematological Alterations

**DOI:** 10.1155/vmi/5750968

**Published:** 2026-10-28

**Authors:** Brishti Barua, Jinnat Ferdous, Sumsoon Naher Tuly, Chandan Nath, Md Abu Sayeed, Md. Mizanur Rahman, Md Ahasanul Haque

**Affiliations:** ^1^ Department of Medicine and Surgery Chattogram Veterinary and Animal Sciences University (CVASU) Chattogram 4225 Bangladesh cvasu.ac.bd; ^2^ School of Veterinary Science The University of Queensland Gatton Queensland 4343 Australia uq.edu.au; ^3^ Department of Microbiology and Veterinary Public Health Chattogram Veterinary and Animal Sciences University (CVASU) Chattogram 4225 Bangladesh cvasu.ac.bd; ^4^ National Centre for Epidemiology and Population Health (NCEPH) The Australian National University Canberra Australian Capital Territory 2600 Australia anu.edu.au

**Keywords:** hematology, PPR, prevalence, risk factors

## Abstract

Bangladesh is an agro‐based country where goat rearing plays a pivotal role in supporting rural livelihoods because of its relatively low investment requirements and high profitability. However, peste des petits ruminants (PPR) remains a major threat to goat health and productivity. This study investigated the clinical and epidemiological characteristics of PPR in goats presented at the Teaching Veterinary Hospital (TVH) of Chattogram Veterinary and Animal Sciences University (CVASU) and in surrounding areas of the Chattogram Metropolitan Area (CMA). A descriptive study was conducted to determine the proportionate prevalence of PPR, assess the frequency of clinical signs, evaluate therapeutic practices, and identify potential risk factors associated with its occurrence. Data from 922 clinically diagnosed PPR cases were retrieved from the TVH database for descriptive analysis. In a separate investigation, 138 suspected PPR cases, including 50 cases at TVH and 88 from diverse sources, were examined clinically and tested using serological and molecular methods. Blood samples were analyzed to determine seropositivity and assess hematological changes, while nasal swabs were subjected to molecular diagnosis. The prevalence of PPR was 21.3%–28.7% based on the descriptive analysis and 21.7% based on molecular testing. The most frequently observed combination of clinical signs was nasal discharge, stomatitis, and diarrhea, which occurred in 17.5% of the cases; sulfadimidine (70.4%) was the most commonly used antimicrobial to treat PPR. Hematological analysis indicated alterations in lymphocyte and neutrophil counts among affected goats. Multivariable analysis revealed that the rainy season, intensive/semi‐intensive rearing systems, Black Bengal breed, and cachectic body condition were significantly associated with an increased risk of disease occurrence. In contrast, sex, body weight, and age showed no significant association. This study highlights the clinical patterns, prevalence, and hematological changes associated with PPR in goats in Chattogram, emphasizing the importance of early detection and targeted control measures to reduce the impact on small ruminant health and rural livelihoods.

## 1. Introduction

The total livestock population in Bangladesh is approximately 464.625 million, including 27.291 million goats [1]. Goats are widely reared by rural households, marginal farmers, children, landless laborers, and economically disadvantaged women who cannot afford to rear cattle. Consequently, Goats are often referred to as the “Poor man’s cow” [2]. It contributes to the national economy by providing high‐quality meat, milk, and skin. It also plays an important role in poverty alleviation, income generation, creation of employment opportunities, and food security [3]. However, infectious diseases remain a major constraint to goat production in Bangladesh. Among these, peste des petits ruminants (PPR), pneumonia, goat pox, enterotoxemia, mastitis, myiasis, tetanus, and ringworm are predominant in goats in Bangladesh [4].

PPR, caused by the PPR virus (PPRV), is an acute, highly contagious, febrile, and transboundary viral disease that primarily affects small ruminants and can also infect wild animals. The disease is widely distributed across Southern Asia, the Arabian Peninsula, the Middle East, and West and Central Africa [5]. PPR is endemic in several countries, including Bangladesh, India, Pakistan, Nepal, Bhutan, Afghanistan, and China [6].

Important risk factors associated with the occurrence of PPR in goats include housing (*p* = 0.008) [7], goat breed (*p* < 0.01) [8–10], age (*p* < 0.001) [8, 9, 11], seasonal variation (*p* < 0.001) [8, 9], rearing system (*p* < 0.05) [10], and sex (*p* < 0.05) [8, 10]. Clinically, PPR in goats is characterized by sudden onset of high fever [12], oculo‐nasal discharge [13], pneumonia [14], stomatitis and profuse diarrhea [15], which may lead to severe dehydration and subsequent death [16]. PPR can cause morbidity and mortality rates of up to 100%, resulting in substantial economic losses. The prevalence of PPR in goats varies considerably among countries, with reported rates of 32.8% in India [17], 45% in the Republic of Niger [18], up to 83% in Turkey [19], 6.8% in Ghana [20], and 10.1%–50.3% in Bangladesh [2, 4, 9, 21–23]. The reported seroprevalence of PPR in goats ranges from 6% to 90.7% in different countries of the world [24–32]. However, in Bangladesh, seroprevalence estimates among goats range from 26.7% to 44% [33, 34]. PPR infection can cause alterations in hematological parameters, including a rapid decline in lymphocyte counts due to the destruction of lymphoid tissues, which may contribute to immunosuppression [35]. In contrast, neutrophil count may increase in response to secondary bacterial infections during PPR infection. Assessment of hematological parameters may therefore support PPR diagnosis and improve diagnostic efficiency, particularly in settings where modern and highly sensitive diagnostic techniques are limited.

Different approaches are used to prevent and control PPR in goats. Vaccination is one of the primary approaches for controlling the disease. In addition, antimicrobial agents, along with symptomatic and supportive therapies, are commonly used in affected goats. However, the extent and pattern of antimicrobial use for the management of PPR have not yet been adequately assessed in Bangladesh.

The objectives of this study were to determine the clinical patterns and prevalence of PPR, seropositivity, and hematological parameters in affected goats, as well as the risk factors associated with PPR prevalence across different sources in selected areas of Chattogram, Bangladesh.

## 2. Materials and Methods

### 2.1. Study Design and Data Collection

The study was conducted in two stages—from January 2011 to December 2015. Suspected cases of PPR from the Teaching Veterinary Hospital (TVH) of Chattogram Veterinary and Animal Sciences University (CVASU) were included in the study, retrospectively, to determine the frequency of clinical signs and evaluate the drugs prescribed for clinically diagnosed PPR cases in goats. The TVH is located in a PPR‐endemic area of Chattogram, Bangladesh, making it an appropriate setting for investigating the clinical manifestations and epidemiological characteristics of the disease. The hospital receives a large number of goat patients daily, especially Black Bengal goats. It is estimated that more than 90% of the goat population in Bangladesh comprises the Black Bengal, while the remainder is Jamnapari and their crosses [36]. Black Bengal goats are highly susceptible to PPR. The TVH maintains a systemic paper‐based disease surveillance data‐recording system and well‐equipped diagnostic facilities, which facilitate the confirmation of PPR cases through clinical signs and laboratory findings. The paper‐based clinical records commonly referred to as “case sheets” contain information on the case registration number and owner’s name and address, presenting complaint, case category, rearing system, breed, age, sex, body condition score (BCS), parity, body weight and clinical signs and affected body systems, rectal temperature, tentative diagnosis followed by confirmatory diagnosis in some cases, prescribed medications, and follow‐up information. In addition, the teaching‐ and research‐oriented environment of TVH facilitates ethical data collection, proper supervision, and evidence‐based clinical management, making the hospital a suitable and reliable source of clinical information on PPR in goats. A PPR case was clinically characterized by coughing, nasal discharge, pneumonia, lacrimation, conjunctivitis, diarrhea, stomatitis, frothy salivation, and fever. A goat exhibiting at least three clinical signs from the aforementioned list was considered a PPR‐suspected case, which was diagnosed by a veterinarian at the TVH. A total of 922 clinically diagnosed PPR cases recorded from January 2011 to December 2015 were included in this study. Besides, three non‐PPR clinical cases of goats were taken as controls (*N* = 2766) for each PPR case (Figure 1). The control cases were selected from non‐PPR goat cases recorded during the same study period as the corresponding PPR cases.

**FIGURE 1:**
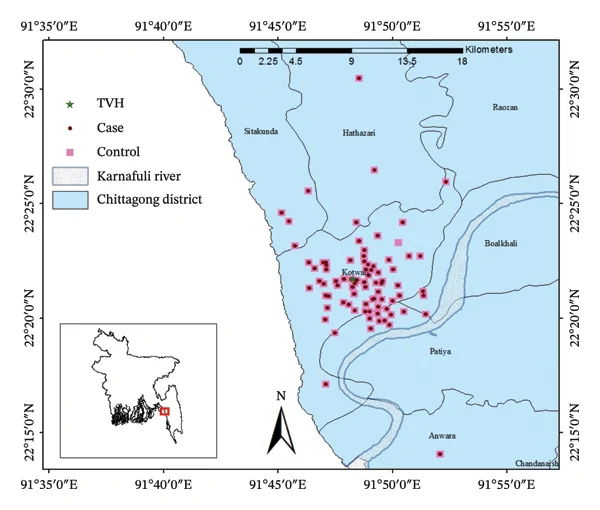
Spatial distribution of PPR and non‐PPR goat cases in the study.

During the second stage of the study, conducted from 15 August 2015 to 30 December 2016, a total of 500 goats infected with PPR and other diseases were included. Of these, 150 goats were from TVH and 350 goats were from seven other sites of CMA, Bangladesh, to estimate both the proportionate prevalence and seropositivity of PPR and to assess the impact of PPR on hematological parameters in affected goats. Of the 350 goat samples from the CMA, 80 and 95 goats were selected from Firingi Bazar and Dewanhat Slaughterhouses, respectively. These are two major slaughterhouses in Chattogram where a large number of goats are slaughtered daily, with animals coming from different geographical areas of the region and considered suitable sites for obtaining a representative sample of the goat population. In addition, two major live animal markets—Bibirhat (*N* = 55) and Sagorika (*N* = 50)—were selected as study sites because they provided suitable surveillance points for monitoring PPR. Then, from three household goat farms (AK Khan household goat farm, Pahartali household goat farm, and Segunbagan household goat farm), 70 goats were included in the study. These farms were located near the TVH of CVASU and practiced small‐scale goat rearing under semi‐intensive management, which is representative of the common goat production system in the area. Goats reared in these farms have a common history of animal movement and contact with other goats through local trading and grazing practices, potentially increasing the risk of PPR transmission (Table 1).

**TABLE 1:** Distribution of PPR‐suspected goats and healthy goats in different study sites.

Sources	Total number of goats (apparently healthy and with PPR)	Number of apparently healthy goats	No. of serum samples from apparently healthy goats	Number of clinically PPR‐suspected goats	No. of serum samples from PPR‐suspected goats
Teaching Veterinary Hospital	150	100	2	50	4
Firingibazar slaughterhouse	80	50	2	30	4
Dewanhat slaughterhouse	95	80	2	15	4
Bibirhat live goat market	55	45	2	10	4
Sagorika live goat market	50	42	2	8	4
A K Khan household goat farm	30	20	2	10	4
Pahartali household goat farm	25	15	2	10	4
Segunbagan household goat farm	15	10	2	5	4
Total	500	362	16	138	32

### 2.2. Biological Sampling

Among the 500 goats, a total of 138 goats exhibited different clinical signs of PPR and were thus selected for biological sampling, including nasal swab and blood samples. Nasal swab samples were used for PPR molecular diagnosis, whereas blood samples were used for PPR antibody evaluation and differential leukocyte counts.

Clinical signs of PPR were recorded through observation and direct physical examination. The numbers of biological samples collected from different sources are shown in Table 1.

Proper personal protective measures were ensured before sample collection. Individual nasal swab samples were collected using sterile cotton swabs and then placed in sterile cryovials containing 5 mL viral transport media (VTM) and labeled with unique identification number and placed in an insulated ice box containing ice packs and immediately transferred to Poultry Research and Training Centre (PRTC) laboratory, CVASU for temporarily stored at −80°C. Then the samples were shifted to SAARC PPR RLDL (South Asian Association for Regional Cooperation Regional Leading Diagnostic Laboratory) in Bangladesh Livestock Research Institute (BLRI), Savar, Dhaka, where the samples were kept at −80°C for further laboratory evaluation.

In addition, 4 mL of blood sample was collected aseptically from the jugular vein of each selected animal using a sterile disposable syringe. A single drop of blood sample was immediately placed on a sterile slide to prepare a direct thin blood smear for hematological examination, and the remaining blood sample in the syringe was poured into a sterile Falcon tube without the addition of anticoagulants for serum separation. The Falcon tubes containing blood samples were placed in a 45° inclined position and transferred to the PRTC laboratory using an insulated ice box. In the laboratory, the Falcon tubes containing blood samples were kept in the refrigerator overnight. Then the serum was separated by centrifugation at 5000 rpm for 15 min. After centrifugation, serum samples were transferred to Eppendorf tubes labeled with the same identification numbers as the Falcon tubes and stored at −20°C until being transported to the SAARC PPR RLDL, BLRI, Savar, Dhaka. In BLRI, the serum samples were stored at −20°C until further evaluation.

### 2.3. Molecular Detection of PPR

For molecular detection of PPR virus, nasal swab samples from 138 suspected goats were tested using pooled samples. A total of 28 pools were prepared: 27 pools consisted of five nasal swab samples each (135 samples), while the remaining pool consisted of three samples, giving a total of 138 samples. Each pool was subjected to PCR for detection of PPR viral RNA. Individual samples from PCR‐positive pools were not tested separately; therefore, the individual‐level positivity was estimated by attributing the five goats represented in each positive five‐sample pool to the positive group. RNA was extracted from the pooled samples according to the manufacturer’s protocol (RNeasy Mini Handbook, 4th edition, April 2006 by Qiagen). Reverse transcription polymerase chain reaction (RT‐PCR) protocol followed by agarose gel electrophoresis technique was used for the detection of PPR viral RNA from nasal swab extracts targeting the nucleoprotein (N) as described by [37]. The oligonucleotide primers used in the study were as follows: NP3 (F) 5′‐TCTCGGAAATCGCCTCACAGACTG‐3′ (position 1232–1255) and NP4 (R) 5′‐CCTCCTCCTGGTCCTCCAGAATCT‐3′ (1583‐1560), and the amplicon size was 351 bp. One‐step RT‐PCR kit (Qiagen Street 1, 40724 Hilden, Germany) was used for the RT‐PCR. After that, agarose gel electrophoresis was performed to analyze the RT‐PCR products, as described by [38].

### 2.4. Serological Testing of PPR

Only 48 serum samples (*N* = 138) were considered for serological analysis using the cELISA technique because of financial constraints. This limited number of serum samples was randomly selected from the list of samples that had low lymphocyte and high neutrophil counts due to lymphopenia and neutrophilia during PPR infection [39]. Among all serum samples, at least four samples and two samples were selected randomly from clinically PPR‐affected goats and apparently healthy non‐PPR goats, respectively, for each study site, maintaining a 2:1 ratio for cELISA testing. The cELISA test was performed as described by [40].

Serum antibodies specific for the PPRV hemagglutinin (H) glycoprotein were analyzed by a commercial PPR cELISA kit produced by BDSL (Biological Diagnostic Supplies Ltd, 25 Main Street, Irvine, KA11 4AQ, Scotland), and the Pirbright Institute for Animal Health was used for the ELISA test to detect the anti‐PPR antibody in the serum samples using the standard process described by [41]. The test protocol was based on competition between the monoclonal antibodies against the hemagglutinin (H) protein of PPR virus and test serum samples to bind with PPR antigen [41].

### 2.5. Blood Smear Preparation

The blood smears were prepared as described in [42]. In brief, a drop of blood was placed at the right end of a clean, grease‐free, smooth, and unbroken glass slide. Then the slide was taken on a piece of paper on the table, held firmly by the forefinger or thumb of the left hand. The even edge of the second slide was placed near the drop of blood toward the middle of the slide. The second slide was drawn toward the drop of blood at an angle of 45°, and the blood was spread along the edge. Keeping the second slide at the same angle, a quick, even push was given with a uniform force toward the other end of the slide. Then the slide was allowed to dry. The dried slides with unique identification numbers were taken to the Physiology Laboratory of CVASU for staining and hematological examination.

### 2.6. Hematological Evaluation

A total of 138 blood films were smeared using Wright’s staining technique as described by [43], placed in a fixed place under a light microscope using immersion oil. Then the cells were identified using a high‐power objective 100x following the parallel stripe method. The counting was continued until 100 cells had been identified. Different types of cells were counted by the tally method. Then percentages of lymphocytes, neutrophils, eosinophils, monocytes, and basophils were recorded.

### 2.7. Statistical Evaluation

Field and laboratory data were entered into Microsoft Excel 2007. Data were cleaned, sorted, coded, and checked for integrity. Afterwards, data were exported to STATA‐IC‐13 *(StataCorp, 4905, Lakeway Drive, College Station, Texas 77845, USA)* for conducting epidemiological analysis.

### 2.8. Descriptive Analysis

We summarized the goat cases diagnosed at TVH, CVASU using frequencies and percentages. The analysis included the seasonal pattern of PPR, the common clinical signs observed, and the drugs most frequently prescribed, which were categorized by their pharmacological action and composition. The overall prevalence of PPR was estimated based on both clinical signs and molecular confirmation, while blood cell counts (lymphocytes and neutrophils) were compared between PPR and non‐PPR cases. Hematological parameters were compared between PPR‐positive and PPR‐negative goats. The distribution of continuous variables was assessed using the Shapiro–Wilk test. Because the hematological variables did not consistently satisfy the assumptions of normality, comparisons between PPR‐positive and PPR‐negative groups were performed using the two‐tailed Mann–Whitney *U* test. Results are presented as mean ± standard deviation (SD), median with interquartile range (IQR), and range. Mean differences between groups with 95% confidence intervals (CIs) were also calculated. Effect sizes were assessed using Cliff’s delta (*δ*), with negative values indicating lower values in PPR‐positive goats and positive values indicating higher values in PPR‐negative goats. A two‐tailed *p* value < 0.05 was considered statistically significant.

### 2.9. Risk Factor Analysis

To identify potential risk factors, individual variables, including season, rearing system, breed, body condition, sex, weight, and age, were initially assessed using Fisher’s exact test. Variables showing significant associations (*p* ≤ 0.05) with PPR cases were subsequently entered into a multivariable logistic regression model. For the analysis, categories were regrouped, including summer with winter versus rainy; Jamnapari/Crossbred versus Black Bengal. We also checked for possible confounding, interactions, and collinearity. The performance of the final model was evaluated with an ROC curve and the Hosmer–Lemeshow test, and the results were expressed as odds ratios with 95% CIs.

## 3. Results

### 3.1. Clinical Patterns, Seasonal Trends, and Therapeutic Management of PPR at TVH

The temporal patterns of PPR cases were evaluated by year and season, while antimicrobials and other supportive therapeutics used for PPR‐positive cases were illustrated in Table 2. The frequency of PPR‐positive cases increased progressively from 21.2% in 2011 to 28.7% in 2015. The highest prevalence of PPR was found in the rainy season (65.6%), followed by the winter season (18%) and summer season (16.4%). Sulfadimidine sodium was the most frequently prescribed antimicrobial against PPR (70.2%), followed by gentamicin (15.4%) and ceftriaxone (4.6%). Among the combined preparations, aminoglycosides with sulfonamide were used more frequently (1.8%) than aminoglycosides with penicillin (0.8%). Antihistamine and saline were the most used (47.3%) supportive drugs in PPR cases, followed by vitamin‐saline (21.2%) and saline only (15.8%). Nasal discharge was recorded as the most frequent clinical sign of PPR (66.30%), followed by diarrhea (59.80%), coughing (50.12%), and stomatitis (50%) (Figure 2).

**TABLE 2:** Year based (2011–2015) and Seasonal distribution of cases and distribution of prescribed drugs against PPR in Goats at TVH (*N* = 922).

Variable	Category	Case/month/drug	PPR positive/treated goats (*n*)	%
Year	2011	713	151	21.2
	2012	763	177	23.2
	2013	728	188	25.8
	2014	752	196	26.1
	2015	732	210	28.7

Season	Winter	November–February	166	18.0
	Summer	March–June	151	16.4
	Rainy	July–October	605	65.6

Antimicrobial treatment	Sulfonamides	Sulfadimidine	647	70.2
	Aminoglycosides	Gentamicin	142	15.4
	Cephalosporin	Ceftriaxone	42	4.6
	Beta lactam	Amoxicillin	35	3.7
	Aminoglycosides and beta‐lactam	Sulfadiazine, sulfadimidine, sulfapyrimidine, and streptomycin	18	1.8
	Fluoroquinolone	Ciprofloxacin	18	1.9
	Tetracycline	Oxytetracycline	13	1.4

Supportive treatment		Antihistamine and saline	436	47.3
		Vitamin and saline	195	21.2
		Saline	146	15.8
		Antihistamine and antipyretic	53	5.7
		Antihistamine and vitamin	51	5.5
		Antipyretic and saline	41	4.50

*Note:* Annual data represent the number and percentage of PPR‐positive goats among goats examined during 2011–2015. Seasonal data represent the number and percentage distribution of the 922 PPR‐positive goats according to month of occurrence: winter (November–February), summer (March–June), and rainy season (July–October). Treatment data represent the antimicrobial and supportive therapies administered to PPR‐positive/clinically affected goats. Percentages for antimicrobial treatment may not sum to 100% or equal 922 because some goats did not receive antimicrobial treatment.

**FIGURE 2:**
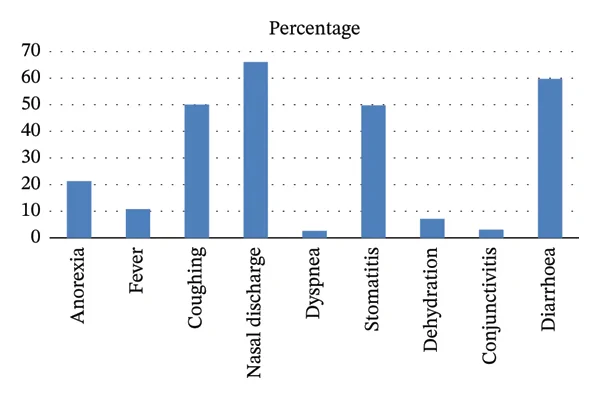
Percentage of goats showing symptoms of clinical PPR in goats at TVH (*N* = 922).

### 3.2. Potential Risk Factors of PPR Cases in TVH (922 Cases, 2766 Noncases)

The univariate analysis revealed that season, rearing system, breed, BCS, and body weight were significantly associated with PPR occurrence, while sex and age were not significant (Table 3). The PPR cases were most significantly (*p* < 0.001) found in rainy season (34.7%), followed by the winter season (25.7%) and summer (11.7%). The goats raised under intensive and semi‐intensive (32.7%) housing systems were significantly (*p* < 0.001) associated with PPR occurrence than free‐ranging and tethering (10.20%). In addition, Black Bengal goats (27.8%) were found to be more susceptible (*p* = 0.015) to PPR than Jamnapari and cross breed (23.9%), and cachectic goats had a significantly higher proportion of cases (36.2%, *p* < 0.001). After adjusting the factors each other, the final logistic model was developed. The odds of PPR were 2.4 times higher during the rainy season in comparison to summer and winter together (95% CI: 1.9–2.9; *p* < 0.001). The odds of PPR was 4.3 times higher among goats reared in intensive and semi‐intensive systems than those goat populations reared in free‐range and tethering systems (95% CI: 3.3–4.5; *p* < 0.001). Goats with cachectic BCS had 5.7 times more likely to be affected by PPR than goats with fair to good BCS (95% CI: 4.5–7.1; *p* < 0.001). However, Black Bengal goat had 1.2 times more likely to be affected by PPR than Jamnapari and other crossbreeds (95% CI: 1.0–1.5; *p* = 0.04). Although body weight demonstrated a significant association with PPR occurrence in univariate analysis, it was excluded from the final multivariable model during automated step‐wise selection as it did not retain statistical significance after adjusting for age, breed, and season due to confounding and collinearity. The full multivariable model demonstrated acceptable discrimination (ROC AUC = 0.655), and the Hosmer–Lemeshow test was statistically significant (*χ* ^2^ = 67.54, df = 8, *p* < 0.001), indicating evidence of lack of fit between the observed and predicted probabilities.

**TABLE 3:** Univariate and multivariate analysis of potential risk factors associated with PPR occurrence in goats presenting at Teaching Veterinary Hospital (2011–2015) (*N* = 3688; Cases = 922, Controls = 2766).

Factor	Category	*N*	Univariate analysis			Multivariate analysis		
			PPR		*p* value	OR	95% CI	*p* value
			Positive (%)	Negative				
Season	Winter	646	166 (25.7)	480	< 0.001	1	—	—
	Summer	1296	151 (11.7)	1145				
	Rainy	1746	605 (34.7)	1141		2.4	1.9–2.9	< 0.001

Rearing system	Free range and tethering	1264	129 (10.2)	1135	< 0.001	1		
	Intensive and semi‐intensive	2424	793 (32.7)	1631		4.3	3.3–4.5	< 0.001

Breed	Black Bengal	1059	294 (27.8)	765	0.015	1.2	1.0–1.5	0.04
	Jamnapari and cross	2629	628 (23.9)	2001		1		

Body condition score	Cachectic	644	233 (36.2)	411	< 0.001	5.7	4.5–7.1	< 0.001
	Fair to good	3044	689 (22.6)	2365		1		

Sex	Male	1732	433 (25.0)	1299	1.000	—	—	—
	Female	1956	489 (25.0)	1467		—	—	—

Body weight (Kg)	≤ 9.5	941	195 (20.7)	746	0.002	—	—	—
	> 9.5 to ≤ 20	1927	511 (26.5)	1416		—	—	—
	> 20 to ≤ 87	820	216 (26.3)	604		—	—	—

Age (Month)	≤ 5 months	935	216 (23.1)	719	0.28	—	—	—
	> 5 to ≤ 18	2000	517 (25.9)	1483		—	—	—
	> 18 to ≤ 60	753	189 (25.1)	564		—	—	—

### 3.3. PPR Infection Status

Of the 28 nasal swab pools tested by PCR, six pools were positive, and 22 were negative. The six positive pools comprised 30 goats (6 × 5), while the 22 negative pools comprised 108 goats (21 pools × 5 goats and one pool × 3 goats). Because individual samples from the positive pools were not tested separately, these 30 goats were classified as positive based on the PCR result of their respective pools rather than individual confirmation. Thus, the estimated pool‐based molecular positivity was 21.7% (95% CI 15.6%–29.4%) among the 138 sampled goats (Figure 3). The molecular detection rate and seropositive rate of PPR‐infected goats are presented in Table 4. The highest positivity was detected among goats from the Bibirhat live goat market (50.0%; 5/10) and Pahartali household farm (50.0%; 5/10), followed by Dewanhat slaughterhouse (33.3%; 5/15), TVH, CVASU (20.0%; 10/50), and Firingibazar slaughterhouse (16.7%; 5/30). No positive cases were detected at the Sagorika live goat market, AK Khan, and Segunbagan household goat farm.

**FIGURE 3:**
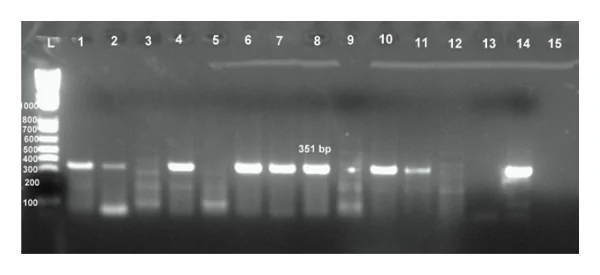
Agarose gel electrophoresis for the detection of PPR targeting nucleoprotein (N) (351 bp). Here, L = DNA ladder, 100 bp plus; Lanes 1–15, samples.

**TABLE 4:** Prevalence of seropositivity of PPR among tested samples in CMA.

Sources	*N*	Apparently healthy goats			Clinical PPR cases				
		*n*	Serum samples, *n*	PPR ab +ve in cELISA *n* (%)	*n*	Serum samples, *n*	PPR ab +ve in cELISA *n* (%)	No of PPR +ve in RT‐PCR (%)	95% CI
TVH	150	100	2	1	50	4	2	10 (20.0)	11.0–33.2
Firingibazar slaughterhouse	80	50	2	0	30	4	2	5 (16.7)	6.9–34.0
Dewanhat slaughterhouse	95	80	2	0	15	4	2	5 (33.3)	14.9–58.5
Bibirhat live goat market	55	45	2	0	10	4	2	5 (50.0)	23.7–76.3
Sagorika live goat market	50	42	2	0	8	4	2	0 (0)	0‐37.2
AK Khan household goat farm	30	20	2	0	10	4	2	0	0‐32.1
Pahartali household goat farm	25	15	2	0	10	4	2	5 (50)	23.66–76.34
Segunbagan household goat farm	15	10	2	2	5	4	2	0	0‐48.9
Total	500	362	16	3 (18.7)	138	32	16 (50)	30 (21.7)	(15.6‐29.4)

*Note:* +ve = positive.

Abbreviation: CI, confidence interval.

The serological test revealed that 3 (18.75%) goats were positive for PPR antibodies among apparently 16 goats. The seropositive rate is the highest in Segunbagan Household Farm 2/2 and TVH 1/2, though the size of the tested serum sample is very small. On the other hand, 32 serum samples from clinically suspected goats were tested by cELISA, of which 16 (50%) tested positive for PPR antibodies.

### 3.4. Lymphocytes and Neutrophils Counts in PPR Goat Cases

The mean and minimum–maximum ranges of lymphocyte (L) and neutrophil (N) counts among PPR‐positive and PPR‐negative goats at the different study sites are presented in Table 5. Overall lymphocyte counts were comparatively lower in PPR affected goats (37.1% in TVH, 41.8% in Firingibazar slaughterhouse, 42.4% in Bibirhat live goat market, 32.2% in Dewanhat slaughterhouse and 38.6% in Pahartali household goat farm than in non‐PPR affected goats (40.2% in TVH, 52.7% in Firingibazar slaughterhouse, 49.2% in Bibirhat live goat Market, 64.5% in Dewanhat slaughterhouse and 41.2% in Pahartali household goat farm).

**TABLE 5:** Distribution of lymphocytes and neutrophils in PPR positive goats/non PPR goats.

Site	PPR (+ve)	Mean		Min‐Max		PPR (−ve)	Mean		Min‐Max	
		L	N	L	N		L	N	L	N
TVH	10	37.1	64.3	30‐45	60‐68	40	40.2	57.2	30‐75	39‐64
Firingibazar slaughterhouse	5	41.8	60.8	34‐45	59‐63	25	52.7	52.6	30‐78	37‐65
Bibirhat live goat market	5	42.4	60.4	37‐45	57‐65	5	49.2	54.8	30‐78	39‐65
Dewanhat slaughterhouse	5	32.2	67	27‐40	65‐68	10	64.5	55.5	38‐78	51‐61
Pahartali household goat farm	5	38.6	60.8	28‐47	56‐65	5	41.2	54.4	30‐78	49‐57
A K Khan household goat farm	0	—	—	—	—	10	47.4	57.2	31‐55	53‐63
Sagorika live goat market	0	—	—	—	—	8	49.6	58.4	47‐53	53‐65
Segunbagan household goat farm	0	—	—	—	—	5	50.8	62.2	43‐57	58‐67

*Note:* Min = Minimum and Max = Maximum; L = lymphocyte; N = Neutrophil.

In contrast, overall neutrophil counts were higher in PPR affected goats (64.3% in TVH, 60.8% in Firingibazar slaughterhouse, 60.4% in Bibirhat live goat market, 67% in Dewanhat slaughterhouse and 60.8% in Pahartali household goat farm) than in non‐PPR affected goats (57.2% in TVH, 52.6% in firingibazar slaughterhouse, 54.8% in Bibirhat live goat market, 55.5% in Dewanhat slaughterhouse and 54.4% in Pahartali household goat farm). A significant difference in hematological profiles was observed between PPR‐positive and PPR‐negative goats. The lymphocyte percentage was significantly lower in PPR‐positive goats than in PPR‐negative goats (median [IQR]: 37.5 [35–43] vs. 51.0 [37.25–54.25]%; Mann–Whitney *U* = 845.5, *p* < 0.001). The mean difference between the groups was −10.66% points (95% CI: −13.95 to −7.44), with a moderate‐to‐large effect size (Cliff’s *δ* = −0.478). Conversely, the neutrophil percentage was significantly higher in PPR‐positive goats than in PPR‐negative goats (median [IQR]: 62.0 [60–66.75] vs. 57.25 [54–60] %; Mann–Whitney *U* = 2745.5, *p* < 0.001). The mean difference was 6.56% points (95% CI: 4.84–8.29), with a large effect size (Cliff’s *δ* = 0.695). These findings indicate a significant shift toward lymphopenia and neutrophilia among PPR‐positive goats (Table 6).

**TABLE 6:** Comparison of leukocyte profiles between PPR‐positive and PPR‐negative goats.

Parameter	PPR‐positive (*n* = 30)	PPR‐negative (*n* = 108)	U	*p* value	Mean difference (95% CI)	Cliff’s *δ*
Lymphocyte (%)	38.20 ± 5.57; 37.5 (35–43)	48.86 ± 13.82; 51.0 (37.25–54.25)	845.5	< 0.001	−10.66 (−13.95 to −7.44)	−0.478
Neutrophil (%)	62.93 ± 3.71; 62.0 (60–66.75)	56.37 ± 5.97; 57.25 (54–60)	2745.5	< 0.001	6.56 (4.84–8.29)	0.695

## 4. Discussion

PPR is one of the most devastating and economically important diseases affecting goats in Bangladesh. The present epidemiological study was conducted at the TVH of CVASU and other selected sites of CMA to determine the prevalence of PPR, identify associated risk factors, clinical signs, and assess the drugs used for its treatment.

In the current study, the overall prevalence of PPR in goats in the CMA was 21.7% among prospective cases, whereas the prevalence among retrospective cases recorded at the TVH ranged from 21.2% to 28.7%. Similar findings were reported in Turkey (20%) [19] and in Bangladesh, including Rajshahi (20.6%) [8] and Jashore (21.3%) [23]. However, Other studies conducted in Bangladesh reported varying PPR prevalence rates: 8.9% [9] and 30.79% [44] in Chattogram, 18.8% in Rangpur, 12.5% in Kushtia [4], 50.3% in Patuakhali [2], and 39% in Thakurgaon [21]. Studies from other countries also reported considerable variation in PPR prevalence, with rates of 6.8% in Ghana [20], 47.9% in Niger [18], 32.8% in India [17], and 51% in Pakistan [45]. The variation in PPR prevalence observed among different studies may be attributable to variations in geographical location, climatic conditions, husbandry practices, animal movement, status of animals, and vaccination and disease control programs, all of which influence the epidemiology and transmission of PPRV [5, 46].

In the current study, approximately half of the serum samples were seropositive, with 50% of samples from PPR‐affected goats and 18.8% from apparently healthy goats testing positive, which could be due to exposure to wild‐type PPR virus or previous PPR vaccination. The present findings are supported by the studies conducted in Bangladesh (49.2%) [47] and in Pakistan (52.9%) [29]. The observed seropositivity was comparable to, but varied from, those reported in previous studies including as 43.3% in Punjab [26], 33%–35% in India [24, 27], 63.4% in Kerala [48], 17.6% in Tibet and China [49], 9% in Afghanistan [25], 15.1% in Saudi Arabia [30], 50.7% in Sudan [50], 45% in Egypt [10] and 59.8% in Northern Ethiopia [51].

The current study identified season as a significant risk factor for the occurrence of PPR (OR = 2.4; Rainy season versus the combined summer and winter season), which is consistent with several studies conducted in Bangladesh [9, 52] and elsewhere [53]. The higher occurrence of PPR during the rainy season may be related to heavy rainfall and environmental conditions that could favor the survival and transmission of the virus [54]. However, few studies identified the winter season as a risk factor for the occurrence of PPR in goats [8, 55]. This may be attributed to dusty and dry winds during winter, which could facilitate the transmission of PPR virus [8]. In addition, the presence of saprophytic organisms in the respiratory tract may predispose goats to pneumonia, potentially increasing their susceptibility to PPR infection [8]. In contrast, another study [56] reported a higher prevalence of PPR in the dry season compared to the wet or rainy season.

The rearing system, with intensive and semi‐intensive rearing systems associated with higher odds of PPR occurrence than free and tethering systems (OR = 4.3). However, this finding differs from that of a study conducted in India, which reported that free‐ranging or tethering systems had a potential influence on the occurrence of PPR in goats [17]. PPR is primarily transmitted through direct contact and respiratory secretions, with transmission facilitated by aerosols and contaminated fomites or surfaces [57–59]. Close contact, particularly in intensive and semi‐intensive rearing systems, further enhances the transmission of the disease. Again, free‐range or tethering systems may have limited access to veterinary care, which could increase their risk of PPR infection. Furthermore, free‐ranging animals frequently move from one place to another and are responsible for the spread of the virus [60].

The present study identified breed as a potential risk factor for PPR occurrence, with Black Bengal goats having slightly higher odds of PPR occurrence than Jamnapari and cross‐bred goats (OR = 1.2, 95% CI 1.0–1.5). This finding is consistent with several studies conducted in Bangladesh [2, 8, 9] and Sudan [10]. Previous studies have reported a higher occurrence of PPR among Black Bengal goats [8, 23, 61–63]. However, the association observed in the present study should be interpreted cautiously and does not necessarily indicate an inherent biological susceptibility of Black Bengal goats to PPR, as differences in management practices, husbandry conditions, exposure patterns, and other factors may contribute to the observed association.

BCS was also identified as a potential risk factor for PPR in goats, with cachectic goats having higher odds of PPR occurrence than goats with fair to good BCS (OR = 5.7), representing a new finding of the current study. However, the finding is biologically plausible because cachectic animals become immunocompromised, which could increase their susceptibility to various infections [64].

The most frequently observed clinical signs among PPR‐affected goats in the present study were diarrhea, nasal discharge, coughing, and stomatitis, which are in line with the previous studies conducted in different countries in the world, including Turkey [19, 39], Egypt [65], Tanzania [66], and Pakistan [54]. These studies also reported nasal discharge, diarrhea, stomatitis, anorexia, coughing, and fever as common clinical signs of PPR. The clinical signs observed in this study may therefore be useful to diagnose PPR in goats in the field condition.

In the present study, the lymphocyte count was found lower in PPR‐affected goats in comparison to healthy goats, which may be due to destruction of lymphoid tissues, as PPR virus has a strong tropism for lymphoid organs [67, 68]; however, this finding was contrary to that reported by [63]. In contrast, the neutrophil count was higher in PPR‐affected goats than in healthy goats. This could be due to having concurrent bacterial infection, which is supported by earlier studies conducted in Kenya [39, 69] and in Bangladesh [12, 70] but was inconsistent with the report of [63].

Sulfonamides and aminoglycosides were the most frequently used antimicrobial drugs against PPR‐affected goats at TVH, primarily to control secondary bacterial infection. Other supportive therapeutics, including antihistamines and saline, were also used to facilitate recovery. The use of sulfonamides in PPR treatment has also been reported by [2, 57, 62] in Bangladesh and in India [71]. These drugs are also comparatively inexpensive, readily available, and effective against a range of bacterial infections. The frequent use of other supportive treatments, including antihistamines, vitamins, and saline for rapid recovery, was also reported in the previous studies conducted in Bangladesh [2, 12, 61].

The current study was conducted to assess the prevalence of PPR among vaccinated and nonvaccinated goats. The prevalence of PPR was 0% among vaccinated goats and 33.3% among nonvaccinated goats, suggesting a possible association between vaccination status and PPR occurrence; however, these findings should be interpreted cautiously given the relatively small sample size [2, 72]. However, this finding should be considered with caution because the current case‐control study included a relatively small sample size.

This study had some limitations in the retrospective cases from TVH. Incomplete hospital records and reliance on clinical signs may have introduced information bias or diagnostic error. In the prospective cases from CMA, the relatively small sample size and limited laboratory testing due to financial constraints reduced the strength of the findings. Additionally, the use of pooled nasal samples may have affected diagnostic accuracy.

## 5. Conclusions

This study highlights that PPR remains an important health challenge in goats, particularly during the rainy season, under intensive or semi‐intensive production systems, and among Black Bengal goats with poor body condition. Vaccination was associated with reduced PPR occurrence, although broader application and further evaluation are needed to confirm its impact. Clinical signs such as nasal discharge, stomatitis, coughing, and diarrhea, together with reduced lymphocyte counts, may support early diagnosis. Supportive treatment with sulfonamides, aminoglycosides, antihistamines, vitamins, and saline was commonly used and may aid recovery by reducing complications. Strengthening vaccination programs, improving hygiene and biosecurity, and promoting better nutritional management may help reduce the burden of PPR in goats.

## Author Contributions

Brishti Barua: conceptualization, data curation, methodology, visualization, writing–original draft, and writing–review and editing. Jinnat Ferdous: data curation, methodology, formal analysis, visualization, and writing–review and editing. Sumsoon Naher Tuly: data curation, methodology, and writing–review and editing. Chandan Nath: data curation, methodology, formal analysis, visualization, and writing–review and editing. Md Abu Sayeed: validation and writing–review and editing. Md. Mizanur Rahman: supervision, validation, and writing–review and editing. Md Ahasanul Haque: funding acquisition, project administration, supervision, validation, and writing–review and editing.

## Funding

This study was funded by the Advanced Study and Research of CVASU. This was funded by a general master′s student. So, no grant number was issued.

## Disclosure

All the authors read and accepted the article. A thesis has previously been published as a preprint (https://dspace.cvasu.ac.bd/bitstream/123456789/1926/1/Brishti_Thesis_updated.pdf) on the website of the library of CVASU [73].

## Ethics Statement

Samples were collected by trained veterinarians using appropriate animal restraint procedures in accordance with established animal welfare principles. No animals were sacrificed specifically for this study. Verbal consent for the study was obtained from the appropriate authority of the Advanced Studies and Research, Chattogram Veterinary and Animal Sciences University (CVASU). In addition, permission was obtained from the Teaching Veterinary Hospital (TVH), CVASU, to access and use the hospital records for the retrospective component of the study. No approval number was issued for this study.

## Conflicts of Interest

The authors declare no conflicts of interest.

## Data Availability

The authors are responsible for sharing all the data based on the findings of the study upon proper request.
